# A Word for the Journal

**Published:** 1879-08

**Authors:** J. F. Miner


### A WORD FOR THE JOURNAL.

BY PROF. J. F. MINER, M. D.

CONGRATULATIONS are due the readers of the new volume of the JOURNAL upon its improved appearance and enlarged scope. For the past many years it has been issued regularly to subscribers, and has been all, and just what, the profession has made it. It now passes into the hands of a corps of editors, who by their united efforts will be able to bestow upon it much labor and thought. It must, however, continue the child of the profession, and be what its care and cultivation make it. The editors are educated, earnest, capable men, and will do their part with faithfulness and fidelity, and if at any time the profession should conclude that there is any defect or want of interest in the JOURNAL, it may also conclude that Buffalo physicians are defective, and not that the editorial staff are inactive or incapable. The JOURNAL, if it is poor and unworthy of support, shows how poor and barren of thought the whole body of the profession in Buffalo and vicinity may be regarded, by those best able to judge. But of the future JOURNAL there can be no doubt. The whole profession, within the scope of its circulation and influence, are ready for its support; all are interested in the practice and progress of medicine.

In withdrawing from the editorial conduct of the JOURNAL, I bespeak for my successors, the support and favor of old friends whose interest and help have been a continual strength to me. This I am sure can not be lessened by change or time, and will be as freely and generously extended to my successors as it has been kindly bestowed upon me.

### TO THE MEDICAL PROFESSION.

The retirement of Dr. Miner from the editorial chair, which he has so long and ably filled, and the strong and earnest words of sympathy expressed in his valedictory, call for a word to the numerous subscribers and patrons by whom the monthly visits of the JOURNAL have been cordially welcomed in the past.

By an arrangement with the late editor, all subscriptions paid to him will be continued until the date of their expiration. All subscribers in arrears will settle with Dr. Miner *up to July 1st.* In such cases the August number will commence a new subscription. In order to adapt the JOURNAL to the demands of the times, the subscription price has been reduced to $2.00 per year, in advance, while the size of the JOURNAL remains the same. It is intended to present bills for subscriptions, and we request a prompt response. An active, live journal, devoted to the interests of the medical profession, can only be maintained by an equally active and live constituency; and we have the confidence that the same earnest wishes for success and active co-operation on the part of the friends of the JOURNAL which have been manifested in the past, will continue with the present management.

The JOURNAL will strive to present a faithful record of the proceedings of the medical societies of Buffalo and vicinity.

We beg the old friends of the JOURNAL to renew their subscriptions promptly, to send new subscribers, to report cases of professional interest, and thus enlarge the field of labor which it is the design of those upon whom its management now depends, assiduously to cultivate.

